# Anterior Petrosectomy vs. Retrosigmoid Approach—Surgical Anatomy and Navigation-Augmented Morphometric Analysis: A Comparative Study in Cadaveric Laboratory Setting

**DOI:** 10.3390/brainsci15020104

**Published:** 2025-01-23

**Authors:** Stefano Signoretti, Francesco Signorelli, Alessandro Pesce, Alberto Delitala, Massimiliano Visocchi

**Affiliations:** 1 Division of Neurosurgery, Department of Head & Neck Surgery, S. Eugenio/CTO Hospital, ASL Roma 2, 00144 Rome, Italy; stefano.signoretti@aslroma2.it; 2 Institute of Neurosurgery, Fondazione Policlinico Universitario A. Gemelli IRCCS, Catholic University, 00153 Rome, Italy; mvisocchi@hotmail.com; 3 Department of Neurosurgery, University of Rome “Tor Vergata”, 00133 Rome, Italy; ale_pesce83@yahoo.it; 4 Division of Neurosurgery, San Carlo di Nancy Hospital, 00165 Rome, Italy; delitalanch@gmail.com; 5 Research Center and Master II Degree Surgical Approaches Craniovertebral Junction, Fondazione Policlinico Universitario A. Gemelli IRCCS, Catholic University, 00153 Rome, Italy

**Keywords:** anterior petrosectomy, petroclival lesions, morphometric anatomical analysis, neuronavigation

## Abstract

Background: Different lateral and postero-lateral cranial approaches to the petroclival region and to the mid-upper brain stem have been described so far, some of which require extensive osseous demolition and possible damage of neurovascular structures. Neuronavigational systems are now extensively available for preoperative planning and intraoperative navigation to assist the surgeons in choosing the optimally invasive approach for each pathology. Herein, we describe a detailed navigation-augmented morphometric analysis to bring insight into the usefulness of an anterior petrosectomy (AP) to handle lesions in the petroclival region. Methods: Eight cadaveric, silicone injected heads were used. A total of 14 approaches (AP, *n* = 8; retrosigmoid, RS, *n* = 6) using a standard microsurgical dissection technique were performed. All specimens had preoperative CT and MRI scans, as well as a post-dissection CT. The neuronavigational system was used for distance measurements, craniotomy sizes and surgical corridor volumes, for each approach. Results: The distance from the skull surface to the petrous apex was significantly shorter in the AP approach when compared with the RS (46.0 ± 1.9 mm versus 71.3 ± 1.8 mm, respectively, *p* < 0.001). Although the craniotomy size was not different, the volume of the surgical corridor was significantly larger with the AP approach (21.31 ± 1.91 cm^3^ vs. 13.39 ± 1.8 cm^3^). The AP approach increased the length of the basilar artery exposure from 6.9 ± 1.5 mm (obtained with a standard subtemporal approach) to 22.1 ± 1.7 mm (*p* < 0.0001). Conclusions: The surgical corridor to the petroclival region achieved by virtue of an AP was significantly larger and featured shorter working distances, resulting in a higher degree of surgical freedom. Although significant individual anatomical variations of fundamental neurovascular and bony structures were found, these difficulties were overcome by careful pre- and intraoperative use of neuronavigation.

## 1. Introduction

The Petroclival region is often considered a “no-man’s land” [1,2] in skull base surgery, presenting extraordinary operative challenges. A variety of approaches have been regularly advocated, either intra- or extradural and ranging from anterior-lateral to posterior routes [2,3,4,5,6]. In the 1980s, Kawase et al. described a subtemporal approach completed by an extradural anterior petrosectomy (AP) to optimize the exposure of the petroclival anatomy [1,7]. However, technical difficulties and considerable intraoperative risks have significantly limited adequate diffusion of the technique. The more familiar and versatile retrosigmoid (RS) approach was, therefore, widely adopted, especially for tumors, despite some well-known disadvantages [3,5,6,8]. Nonetheless, relentless technological progress along with the evolution of neuronavigational systems (NS), determined a true step forward, allowing detailed preoperative planning and precise intraoperative anatomical definition, especially in skull base approaches. In the present cadaveric study, we compare the microsurgical anatomy exposed by the AP and the RS approaches through a morphometric analysis using the routinely used intraoperative navigational technique. The volumes of the surgical corridors and the respective areas of surgical exposure of several anatomical target points were analyzed with the following aims:

1. Measuring anatomical distances, areas and volumes of the surgical pathway, comparing the two approaches;
2. Determining the volume of petrous apex removal to allow for the optimal exposure of the posterior cranial fossa (PCF).

The influence of the head position was also studied in order to define the related surgical anatomy variation.

## 2. Materials and Methods

### 2.1. Laboratory Setup

The cadaveric prosecutions were performed in the cadaveric dissection laboratory at the Medical College of Virginia. A microsurgical laboratory equipped with an operating microscope (Moeller-Wedel MMS 900, Wedel, Germany), a navigational system and image fusion software (BrainLab, Kirchheim, Germany) were used in this study. Embalmed (*n* = 4) and fresh (*n* = 4) cadaver heads were used for this study. We used cadavers of individuals who died between 24 and 48 h prior in nontraumatic circumstances and without relevant intracranial pathological conditions (5 male and 3 female). All specimens were silicone injected and stored in 66% ETOH. A total of 14 approaches (AP *n* = 8; RS *n* = 6) were performed. All 8 specimens underwent preoperative CT and MRI scans. Post-dissection CT scans were performed as well, to evaluate and measure the extent of the drilling, and the volume of the surgical corridor, for both approaches.

Dissections were performed in a realistic fashion, avoiding excessive drilling and unnecessary risks so as to mimic real operative settings.

Every effort was made to comply with all local and international ethical guidelines and laws concerning the use of human cadaveric donors in anatomical research.

### 2.2. Navigational Data Acquisition and Volumetric Measurements

Navigational system image was used for anatomical measurements of the petrous bone, petroclival region and craniotomy sizes, using external markers and intraoperative registration. Using image fusion software, preoperative CT/MRI scans were merged with post-dissection CT images for volumetric assessment. The AP and standard suboccipital RS approaches were compared. The surgical distances between pertinent intracranial structures and a pre-determined cranial point on the surface of the skull (cranial entry point), which reflected the main axis to the target, were also measured. This “cranial entry point” was at the root of the zygoma on the temporal bone for the AP and at the angle between the transverse and the sigmoid sinuses on the occipital bone for the RS approach. In addition to multiple distance measurements, craniotomy areas and surgical corridors for each approach were measured and compared. Furthermore, we used a modified version of the rhomboid construct described by Day et al. [9] for the landmark measurements of the petrous apex. Using navigational software, the angle between the arcuate eminence (AE) and the internal auditory canal (IAC) was obtained.

### 2.3. Surgical Approaches

#### 2.3.1. Transtentorial Transpetrosal Approach

**Figure 1:**
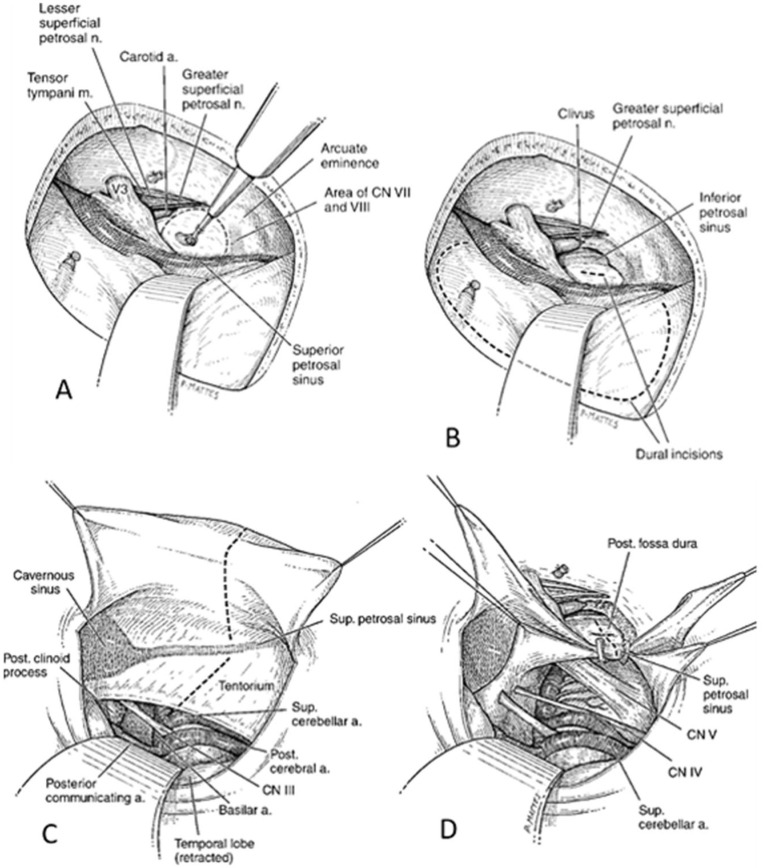
Extradural drilling of the petrous bone and dural incision with exposure of the middle fossa floor dura, the arcuate eminence (AE) and the greater (GSPN) and lesser superficial petrosal nerves, intra-petrous carotid artery and tegmen tympan (Panel **A**,**B**). Incision and retraction of the tentorium with exposure of the pontomedullary junction, V, VI, VII and VIII cranial nerves, the basilar artery, the posterior cerebral (PCA), the superior cerebellar artery (SCA), and the anterior inferior cerebellar artery (AICA) (Panel **C**,**D**).

The steps of this approach are illustrated in Figure 1. Specimens were held in place in a Mayfield head holder, fixed to the laboratory-operating table. The cadaveric head was positioned in a 90° lateral position with the vertex dropped 10° to 15° towards the floor to improve visualization of the clival region. A small question-mark-shaped incision was made, and the scalp was elevated in two layers and reflected anteriorly. The temporalis fascia and muscle were incised along the margin of the skin incision. A second incision along the main axis of the muscle was made to create a posterior “vascularized” musculofascial flap to help later in the closure. The musculofascial flaps were elevated subperiosteally and reflected anteriorly, exposing the root of the zygoma. A small craniotomy centered over the zygomatic root was carried out. The bony margin along the inferior edge of the craniotomy was drilled away to avoid any lip that would prevent a flat angle of view along the middle cranial fossa. The middle fossa floor dura was elevated in a posterior to anterior direction to avoid stretching of the greater superficial petrous nerve as about 15% of geniculate ganglia are dehiscent in the floor of middle fossa with risk of facial nerve damage. The first landmark was generally identified at this stage; however, the appearance of arcuate eminence (AE) can be difficult to recognize, reason why navigational system was used to confirm the position. Further dural elevation unveiled the greater (GSPN) and lesser superficial petrosal nerves, intra-petrous carotid artery and tegmen tympani. The horizontal portion of the petrous segment of the internal carotid artery (ICA) was often visible throughout the bony floor of the middle fossa. The middle meningeal artery was exposed at the foramen spinosum and then divided to permit retraction of the middle fossa floor dura. Proceeding with dural dissection in an anterior medial direction, the V3 nerve and the trigeminal ganglion were exposed, constituting the anterior margin of the dural elevation. However, in some specimens, this area was rather small and needed supplemental drilling around the foramen ovale to mobilize the nerve and to expose the very tip of the petrous pyramid. Before starting drilling, the position of the internal acoustic canal (IAC) was estimated as lying deep to the bisect axis of the angle between the axes of GSPN and AE. The navigational device was used to verify the location of IAC and the cochlea. The cochlea lies deep in the premeatal triangle, which is limited by the carotid genu, geniculate ganglion, and by the medial margin of the IAC. The AP was performed according to the following landmarks: (1) petrous apex (very often the nerve was retracted anteromedially to gain access to the petrous tip under the trigeminal complex in the Meckel’s cave); (2) the GSPN–V3 intersection; (3) arcuate eminence; (4) geniculate ganglion. The petrous bone was drilled entirely extradurally to the level of the inferior petrosal sinus (IPS). After drilling, the tentorium was cut and retracted. The superior petrosal sinus (SPS) was divided after ligation at its medial aspect to preserve the venous drainage from the petrosal vein. The tentorium was then incised and retracted using silk sutures. The dural opening, once the AP was completed, exposed the pontomedullary junction, V, VI, VII and VIII cranial nerves, the basilar artery, the posterior cerebral (PCA), the superior cerebellar artery (SCA), and the anterior inferior cerebellar artery (AICA).

#### 2.3.2. Retrosigmoid Approach

**Figure 2:**
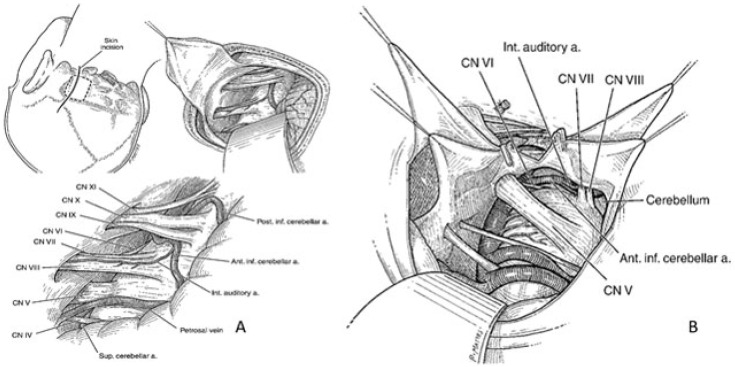
Retrosigmoid craniotomy and retraction of the cerebellum to gain access the neurovascular structures of the cerebello–pontine angle and the petroclival region (Panel **A**,**B**).

The surgical steps of this approach are illustrated in Figure 2 (panel A). A vertical incision was centered 2 cm medial to the mastoid process. A small craniotomy was performed from an initial burr-hole placed just below the transition between the transverse and the sigmoid sinuses. Stellate dural incision provided superior, lateral, and inferior flaps that were held back with sutures. The cerebellum was then retracted to gain access to the neurovascular structures of the cerebello–pontine angle and the petroclival region.

### 2.4. Data Analysis

For calculation of measurements, angles, areas and volumes, we used the software provided by the navigational device. Statistical analysis was performed using paired *t*-test included in the StatView^®^ 5.0 software (Abacus Concepts Inc., Mountain View, CA, USA). All measurements are expressed as average ± standard error of mean (SEM).

## 3. Results

### 3.1. Morphometric Analysis

#### 3.1.1. Target Distances

The surgical distances between the pertinent intracranial structures and the pre-determined cranial point on the surface of the skull (cranial entry point) are expressed in Table 1 and Table 2. The distance from the skull surface to the petrous apex was significantly shorter when performing an AP rather than an RS approach (46.0 ± 1.89 mm versus 71.3 ± 1.8 mm, respectively) (*p* < 0.001). Equally, the distance from the skull surface to the internal auditory meatus was on average significantly shorter for the AP than for the RS approach (*p* < 0.05) (37.4 ± 1.9 mm vs. 47.1 ± 1.9 mm). These two landmarks were chosen since they delineate the anteromedial and the posterolateral limits of the petroclival region. Overall, the surgical distances defined by the AP were shorter when compared to the RS approach.

#### 3.1.2. Surgical Freedom

The average craniotomy size for the AP measured 1271.85 ± 131.71 mm^2^, while the RS approach was 744.03 ± 100.16 mm^2^. Although the craniotomy size was not significantly different, the volume of the surgical corridor was significantly larger for the AP than for the RS approach (21.31 ± 1.91 cm^3^ vs. 13.39 ± 1.8 cm^3^). By virtue of the larger surgical pathway, combined (on average 37% larger for the AP than RS) with the shorter working distance (on average 35% to the apex for the AP compared to RS), resulted in a higher degree of surgical freedom. The best degree of surgical freedom was obtained by removing a bony volume of approximately 2.83 ± 0.80 cm^3^ from the petrous apex.

#### 3.1.3. Exposure of Basilar Artery and Clivus

Exposure of the basilar artery and clivus after the subtemporal-, subtemporal-transtentorial and subtemporal-transtentorial after AP is illustrated in Table 3. Adding the AP maximized the exposure of the basilar artery and clivus (*p* < 0.05). After AP, 22.1 ± 1.7 mm of basilar artery measured from the basilar tip were exposed, compared to 6.7 ± 1.5 mm and 13.1 ± 1.9 mm from a standard intradural subtemporal approach and a subtemporal transtentorial approach, respectively. On average, the subtemporal approach with tentorial division exposed approximately half (58%) of the basilar artery, exposed by adding the AP (*p* < 0.0001). In terms of clival exposure, a 44% increment was obtained by adding the AP to the subtemporal transtentorial approach. The clivus was exposed at a length of 24.3 ± 1.4 mm with AP. Clival exposure reached by the subtemporal measured 3.4 ± 0.6 mm, which increased to 11.4 ± 1.2 mm using the transtentorial route (*p* < 0.05). Interestingly, there was no correlation between the length of the basilar artery exposure and the volume of the petrous bone when drilled (R^2^ = 0.12; *p* = 0.79); similarly, no correlation was shown between the petrous drilling and the extent of clivus exposure (R^2^ = 0.222; *p* = 0.238).

#### 3.1.4. Landmarks for Petrous Apex Removal

Table 4 summarizes the anatomical morphometric measurements between key surgical landmarks for safe petrous apex drilling. The average distance from the end of the arcuate eminence (AE) along the petrous ridge to the geniculate ganglion (GG) was 12.8 ± 1.9 mm. The distance from the GG to the greater superficial petrous nerve (GSPN)–V3 intersection measured 16.8 ± 1.8 mm. The length of the GSPN–V3 intersection to the petrous apex averaged 18.7 ± 1.8 mm. Finally, the distance along the petrous ridge from the petrous apex to the AE averaged 49.9 ± 2.1 mm.

## 4. Discussion

Surgery around the petroclival region has always been considered challenging, carrying considerable risks. In the 1960s, Charles Drake proposed the subtemporal transtentorial approach for aneurysms of the basilar artery [9]. Ten years later, Bochenek and Kukwa [10] described an extended middle fossa approach to the CPA for large vestibular schwannomas. In 1985 and 1991, Kawase reported on the treatment of lower basilar aneurysms and petroclival tumors by means of a middle fossa approach and the extensive drilling of the anterior pyramidal bone [7,8,9,10,11]. The drilling of the rhomboid fossa during the Kawase approach allows access to the Meckel cave, the ventral brainstem above the IAC, and the petroclival junction. The Kawase approach offers the advantages of an extradural procedure, minimizing the effects of brain retraction and injury to the vein of Labbe, and it can be performed standalone (e.g., for pons cavernomas) or as part of exposing more extended skull base corridors such as the transtentorial middle fossa or the presigmoid posterior-middle fossa combined approaches [12].

The retrosigmoid craniotomy (also known as lateral suboccipital) provides a wide, safe and rapidly accessible corridor that is a reliable option for the vast majority of posterior fossa operations. It has a central role in the neurosurgical armamentarium and, due to its extraordinary versatility, the relatively high degree of posterior fossa visualization and the possibility of hearing preservation, and it is the preferred route for addressing lesions predominantly located in the cerebellopontine angle (CPA) or arising from the lateral brainstem or internal auditory canal (IAC) in patients with useful hearing. Additionally, it allows access to the petroclival junction and the foramen magnum. However, it can require prolonged cerebellar retraction and offers limited visualization of the internal auditory canal without intradural, transmeatal drilling [13].

Anatomic dissections play an irreplaceable role in the training of surgeons, especially when approaching complex anatomic regions such as the skull base. During dissections, as well as in a clinical setting, the aid of image guidance systems provides the surgeon with constant orientation in the surgical field, thus increasing the accuracy and safety of the approach [14,15].

In the present cadaveric study, we performed a detailed morphometric analysis of the petroclival region, comparing a standard postero-lateral approach to lateral routes. We presented morphometric data concerning the distances between important landmarks lying on the surface of the middle fossa: our findings confirmed what was previously reported [16,17,18,19]. In addition, we provide new data concerning the distances between the surface of the skull and crucial intracranial bony and neurovascular landmarks. We measured the volume of the surgical corridors and the area of the craniotomies for both approaches.

It was also evident during our study that the volumes of bony removal were variable across the specimens. This is in agreement with recent studies and reflects the variations in bony anatomy.

Our CT quantitative analysis yielded similar volumes of bone removal when compared with other studies [20,21], although the substantial interindividual difference was probably due to the fact that half of the specimens were of the female gender. Females have smaller surgical corridors, particularly in the anterior area of the petrous bone [22]. Moreover, we used a more “conservative” drilling strategy with the aim of mimicking real operative settings rather than maximizing the cadaveric anatomic exposure of the adjacent structures for iconographic purposes. In this regard, when performing the retrosigmoid approach, we spared the posterior semicircular canal nor did we excessively retract the cerebellum.

Although the craniotomy size was similar, at least from a statistical perspective, the volume of the surgical corridor and the distance to hypothetical targets were significantly different. The AP provided a shorter pathway and offered a more generous surgical corridor when compared to RS. The larger surgical corridor was not dependent on the craniotomy size or the degree of brain retraction. The combination of a shorter working distance with a larger surgical corridor determines a greater surgical freedom, convenient for both tumor dissection and vascular repair.

### 4.1. Creating a Surgical Corridor by the Removal of the Petrous Apex

The portion of the petrous apex hidden by the trigeminal ganglion ranged from 4.7 to 16.3 mm. Therefore, the surgical space obtained by the AP may vary according to the size of the pyramidal apex from one specimen to another.

In the present study, we slightly modified the geometric construct proposed by Kawase [1] and Diaz et al. [9].

The modified rhomboid construct was first anatomically defined and then confirmed with the aid of our neuronavigation system, using four landmarks, located within the cranial fossa: (1) petrous apex, (2) intersection of the GSPN and V3, (3) geniculate ganglion, and (4) the AE along the petrous ridge. The removal of bone surrounded by these four landmarks to the level of the IPS created a bony fenestration and corridor of 2.83 ± 0.28 cm^3^, which was free of neurovascular structures. In the geometrical construct defined by Day et al. [18], the removal of bone along the petrous ridge was limited to the portion between the AE and the trigeminal impression leaving the apical petrous bone inferior to the trigeminal ganglion in place. As a result, they could expose an area of 2.9 cm^2^ of posterior fossa dura. Compared to the original work by Kawase, our extended drilling located supero-lateral to the IAC resulted in a larger corridor [11]. The temporal bone fenestrations originally described by House [23] and subsequently by Hitselberg [24] and Day [25] were similar with respect to temporal bone removal, compared to the original Kawase approach, leaving the apical petrous bone under the TN in place.

### 4.2. Comparison Between Anterior Petrosectomy and the Retrosigmoid Approach

In 1985, Kawase et al. were the first to describe an extradural MCF technique, which included the partial resection of the pyramidal bone to approach midbasilar aneurysms [7]. Our results showed that the drilling of the bony area encircled by the GSPN and the TN anteriorly, the carotid artery and the cochlea laterally, the IAC and the semicircular canals posteriorly, was rather safe, lacking neurovascular structures. Bone removal exposed the PCF and the CPA from above and further widened by dividing the tentorium.

The major difference between the AP and the RS approach was that the former relies on an extradural corridor, whereas the latter was very dependent on the cerebellar retraction which renders the RS approach a narrower and longer corridor to reach lesions located in the petroclival area.

According to our morphometric evidence, AP represents the shortest surgical corridor from the cranial surface to the upper and mid-basilar artery, with a better safety-effectiveness profile especially when compared to purely transpetrosal approaches that permanently destroy the cochlea and labyrinth [26,27].

### 4.3. Final Considerations on the Use of a Neuronavigation System

Compared to previous cadaveric investigations, the optimization of bone fenestration and surgical corridors was possible by virtue of neuronavigation, which allowed the localization of critical neurovascular structures. Navigational cadaveric data may result in great assistance being easily comparable with intraoperative navigational data.

However, in some cadavers, we found that bone might present important vascular issues, especially in the clivus, and that dura in this region may harbor wide venous lakes, possibly causing intraoperative bleeding which is difficult to control in live surgery. Concerning AP, possible disadvantages may be the complex anatomy of the neurovascular structures and anatomical landmarks with significant inter-individual variability, to which, the use of neuronavigational systems, has an impressive facilitatory effect.

Navigation was useful in identifying arcuate eminence and verifying the internal auditory canal and the cochlea. In middle fossa surgery, the zygomatic root is also a good anatomical reference after the skin incision. In most cases, both the foramen ovale and foramen spinosum are encountered deep in the zygomatic root. Moreover, the main axis of the internal auditory canal projects toward the zygomatic root. This can be useful in a clinical setting.

Further, in clinical settings, neuronavigation can be useful to avoid CSF leaks by means of preoperative CT volumetric analysis of temporal bone air cells. In more detail, factors that may be associated with postoperative CSF leakage included the air cells of the petrous apex, patterns of pneumatization in the temporal bone and thinning of the tympanic tegmen [28].

Our study has some limitations. Although we did our best to mimic a realistic operative setting, several clinical factors such as bleeding, brain pulsations, CSF presence and cranial nerve function, cannot be replicated in a cadaver lab setting. Moreover, anatomic distortions resulting from the tumor mass effect cannot be accounted for.

## 5. Conclusions

According to the literature data and personal experience, in the present cadaveric investigation, we confirmed that the use of accurate neuronavigational systems increases surgeons’ anatomical confidence, especially when facing narrow and deep surgical corridors. CT scans with bone windows will allow for accurate confirmation of the intraoperative landmarks. The complex surgical anatomy is better managed by the use of navigation focused on bony landmarks, especially during the late stages of dissection. Moreover, it could shorten the learning curve of surgeons approaching skull base pathology.

## Figures and Tables

**Table 1:** Anterior petrosectomy: distances from a fixed point on the skull surface to several intracranial structures SEM = Standard error of mean. Fixed point on the skull surface, which is along the main surgical axis for this approach: the superior border of the zygomatic root at its medial point, halfway between the temporal process of the zygomatic bone and the zygomatic process of the temporal bone.

	Mean ± SEM	Range
**F. Spinosum**	30.8 ± 1.09	25.6–34.5
**F. Ovale**	35.4 ± 0.99	29.4–38.9
**Petrous apex**	46.0± 1.80	36.1–53.2
**AE**	22.9± 2.29	19.2–25.8
**IAC**	37.4 ± 1.70	30.0–46.7
**CN III**	61.8 ± 2.53	51.4–70.8
**CN V**	48.7 ± 1.43	40.1–54.5
**Basilar Tip**	62.4 ± 2.13	46.2–68.0
**AICA**	63.11 ± 1.54	57.8–70.4
**ICA**	34.7± 1.67	24.9–39.7
**IPS**	48.8± 1.54	46.1–53.7
**PCP**	53.6 ± 1.67	44.6–60.8

**Table 2:** Retrosigmoid approach: distances from a fixed point on the skull surface to several intracranial structures. SEM = Standard error of mean. Fixed point on the skull surface, which is along the main surgical axis of this approach: junction between lateral and sigmoid sinus on occipital bone.

	Mean ± SEM	Range
**IAC**	47.2 ± 1.91	38.6–51.0
**F. Jugular**	48.3 ± 2.39	36.8–52.7
**CN V**	5.1 ± 2.12	46.4–61.1
**CN VII-VII**	52.3 ± 1.91	43.9–58.3
**AICA**	65.2 ± 2.11	59.4–74.4
**Clivus**	80.8 ± 2.45	86.6–69.3
**Apex**	71.3 ± 4.6	63.4–75.1

**Table 3:** Exposure of basilar artery and clivus after a subtemporal-, transtentorial subtemporal-, or anterior petrosectomy approach. SEM = Standard error of mean.

Approach	Basilar Artery (mm)	Clivus (mm)
**Subtemporal**	6.8 ± 3.2	3.4 ± 1.7
**Subtemporal-Transtentorial**	13.2 ± 4.3	11.4 ± 3.3
**Anterior Petrosectomy**	22.1 ± 4.8	24.3 ± 3.9

**Table 4:** Morphometric analysis of geometric construct, used to drill the anterior temporal bone. SEM = Standard error of mean. GSPN, greater superficial petrosal nerve; CN–V3, trigeminal nerve, submandibular branch.

Distance (mm)	Mean (±SEM)
**Arcuate eminence to petrous apex**	49.9 ± 2.05
**Petrous apex to GSPN–V_3_ intersection**	18.7 ± 1.76
**GSPN–V_3_ intersection to geniculate ganglion**	22.1 ± 4.8
**Geniculate ganglion to arcuate eminence**	

## Data Availability

All the data generated in the present study were included in this paper after anonymization.
